# A 3-D Finite-Element Minipig Model to Assess Brain Biomechanical Responses to Blast Exposure

**DOI:** 10.3389/fbioe.2021.757755

**Published:** 2021-12-17

**Authors:** Aravind Sundaramurthy, Vivek Bhaskar Kote, Noah Pearson, Gregory M. Boiczyk, Elizabeth M. McNeil, Allison J. Nelson, Dhananjay Radhakrishnan Subramaniam, Jose E. Rubio, Kenneth Monson, Warren N. Hardy, Pamela J. VandeVord, Ginu Unnikrishnan, Jaques Reifman

**Affiliations:** ^1^ Department of Defense Biotechnology High Performance Computing Software Applications Institute, Telemedicine and Advanced Technology Research Center, United States Army Medical Research and Development Command, Fort Detrick, MD, United States; ^2^ The Henry M. Jackson Foundation for the Advancement of Military Medicine, Inc., Bethesda, MD, United States; ^3^ Department of Mechanical Engineering, The University of Utah, Salt Lake City, UT, United States; ^4^ Department of Biomedical Engineering, The University of Utah, Salt Lake City, UT, United States; ^5^ Department of Biomedical Engineering and Mechanics, Virginia Tech, Blacksburg, VA, United States; ^6^ Center for Injury Biomechanics, Virginia Tech, Blacksburg, VA, United States

**Keywords:** blast-induced traumatic brain injury, vasculature, brain biomechanical responses, shock tube, blast exposure, finite-element model

## Abstract

Despite years of research, it is still unknown whether the interaction of explosion-induced blast waves with the head causes injury to the human brain. One way to fill this gap is to use animal models to establish “scaling laws” that project observed brain injuries in animals to humans. This requires laboratory experiments and high-fidelity mathematical models of the animal head to establish correlates between experimentally observed blast-induced brain injuries and model-predicted biomechanical responses. To this end, we performed laboratory experiments on Göttingen minipigs to develop and validate a three-dimensional (3-D) high-fidelity finite-element (FE) model of the minipig head. First, we performed laboratory experiments on Göttingen minipigs to obtain the geometry of the cerebral vasculature network and to characterize brain-tissue and vasculature material properties in response to high strain rates typical of blast exposures. Next, we used the detailed cerebral vasculature information and species-specific brain tissue and vasculature material properties to develop the 3-D high-fidelity FE model of the minipig head. Then, to validate the model predictions, we performed laboratory shock-tube experiments, where we exposed Göttingen minipigs to a blast overpressure of 210 kPa in a laboratory shock tube and compared brain pressures at two locations. We observed a good agreement between the model-predicted pressures and the experimental measurements, with differences in maximum pressure of less than 6%. Finally, to evaluate the influence of the cerebral vascular network on the biomechanical predictions, we performed simulations where we compared results of FE models with and without the vasculature. As expected, incorporation of the vasculature decreased brain strain but did not affect the predictions of brain pressure. However, we observed that inclusion of the cerebral vasculature in the model changed the strain distribution by as much as 100% in regions near the interface between the vasculature and the brain tissue, suggesting that the vasculature does not merely decrease the strain but causes drastic redistributions. This work will help establish correlates between observed brain injuries and predicted biomechanical responses in minipigs and facilitate the creation of scaling laws to infer potential injuries in the human brain due to exposure to blast waves.

## Introduction

Due to the lack of clinical data to assess the effects of blunt and blast loads to the human head, the prevailing approach is to study such phenomena in animal models and then use “scaling laws” to project observed injuries in the animal brain to the human brain (Zhu et al., 2013a; Jean et al., 2014; Wu et al., 2021). Recently, Wu et al. (2021), used such an approach to infer the effects of blunt impact to the human head by establishing correlates in an animal model between observed brain injuries and the associated predicted biomechanical responses (Wu et al., 2021). Such an approach can also be used to investigate the effects of the interaction between explosion-induced blast waves and the human head, which is suspected to cause primary blast injury to the brain (Hicks et al., 2010; Bryden et al., 2019). In this case, experiments to identify potential brain injuries, or changes in the concentration of putative protein brain markers induced by a blast exposure, often involve the use of animal models, such as rats, mice, and minipigs, exposed to blast waves in a laboratory shock tube (Bauman et al., 2009; Sajja et al., 2015; Arun et al., 2020). In contrast, the prediction of the resulting biomechanical responses throughout the animal brain often involves the development of three-dimensional (3-D) finite-element (FE) mathematical models (Zhu et al., 2013b; Kalra et al., 2017; Unnikrishnan et al., 2019).

Recently, our team developed a 3-D high-fidelity FE model of a rat head that accounts for the cerebral vasculature and uses rat-specific material properties characteristic of the high strain rates observed in blast exposures to represent the response of brain tissues and the vasculature (Unnikrishnan et al., 2019). Our simulations showed that incorporation of the vasculature reduces the peak strain in the rat brain by as much as 33% and that the use of rat-specific material properties, instead of using those of humans, leads to a three-fold increase in the predicted strain. Such a high-fidelity model does not exist for pigs, and it is needed because before using scaling laws to infer brain injuries in humans, we should first validate them between two species, which requires exposing animals to blast waves to observe brain injuries and the ability to accurately predict the associated biomechanical responses.

Zhu et al. (2013b) and Kalra et al. (2017) have separately developed FE models to predict the biomechanical responses of blast exposure to a pig’s head. However, their models do not include soft tissues around the skull, which influence how the blast wave interacts with the head and loads the brain, nor do they represent the cerebral vasculature, which is known to increase brain stiffness (Zhu et al., 2013b; Kalra et al., 2017). In addition, they modeled the brain as an elastic, linear-viscoelastic material, whereas high-strain-rate experiments performed on pig brain-tissue samples showed that a hyperelastic, linear-viscoelastic material model is necessary to capture the brain’s nonlinear behavior (Rashid et al., 2013).

To overcome these limitations, here we developed a high-fidelity FE model of a Göttingen minipig head by including a detailed geometry of the head, accounting for the cerebral vasculature network, and using species-specific material properties for the brain tissue and vasculature. We obtained magnetic resonance imaging (MRI) scans of the brain and dura as well as computed tomography (CT) scans of the skull and soft tissue to construct the model geometry and develop a FE mesh of a Göttingen minipig head. In addition, we acquired micro-computed tomography (µCT) images of the vasculature network, generated a 3-D vasculature FE mesh, and integrated it into the FE mesh of the minipig head. Then, we characterized the material properties of brain tissue and vasculature of Göttingen minipigs at low and high strain rates, and incorporated these properties as a hyperelastic, linear-viscoelastic FE model of the minipig head. To validate the model predictions, we exposed Göttingen minipigs to a blast overpressure (BOP) of 210 kPa in a laboratory shock tube, measured brain pressures, and compared them with the model-predicted pressures in the brain. Once validated, we used the minipig head model to investigate how the blast wave interacts with the head and loads the brain. Finally, we quantified the extent to which the representation of the vasculature influences the biomechanical responses in the brain tissues by comparing the model-predicted biomechanical responses using models with and without vasculature.

## Methods

### Shock-Tube Experiments

#### Animal Setup

We used five 23- to 25-week-old male Göttingen minipigs (Marshall BioResources, North Rose, NY) weighing 10.4 ± 0.3 kg [mean ± standard deviation (SD)]. We chose to use a minipig because its brain development, immunologic response, and histologic and vascular anatomy are similar to that of a human (Swindle, 1998; Vodička et al., 2005). Moreover, we chose Göttingen over Yucatan minipigs as they have thinner skulls (4.8 ± 1.2 mm) compared to humans (6.9 ± 1.2 mm) (McElhaney et al., 1970), whereas the Yucatan minipig skulls are thicker by approximately 1.7 mm (8.6 ± 1.1 mm).

We implanted pressure sensors in the brain to record the intracranial pressures during blast exposures. For each animal, we placed a catheter (20 gauge) in a saphenous vein in a hind leg and anesthetized the animal using boluses of propofol (2.0 mg kg^−1^) and fentanyl (0.005 mg kg^−1^). When necessary, we intubated the animal to allow for ventilation using either a ventilator or an Ambu bag. We maintained the anesthesia with a combination of propofol (2.0–4.4 mg kg^−1^ hr^−1^), fentanyl (0.003–0.005 mg kg^−1^ hr^−1^), and midazolam (0.4–0.7 mg kg^−1^ hr^−1^), with propofol boluses (0.5–1.0 ml) given as needed. Once the animal was stable, we shaved the incision site and administered subcutaneous bupivacaine (0.5–1.0 ml, 0.5%), a local anesthetic.

We used a nuchal approach to implant the pressure sensors (model 060; Precision Measurement Company, Ann Arbor, MI) in the brain. With this technique, we preserved the integrity of the dorsal aspect of the skull that was directly exposed to the BOP during testing. For the duration of surgery, we positioned the animal so that the nose was directed downward, which enabled us to access the caudal aspect of the skull while minimizing the loss of cerebrospinal fluid.

**FIGURE 1:**
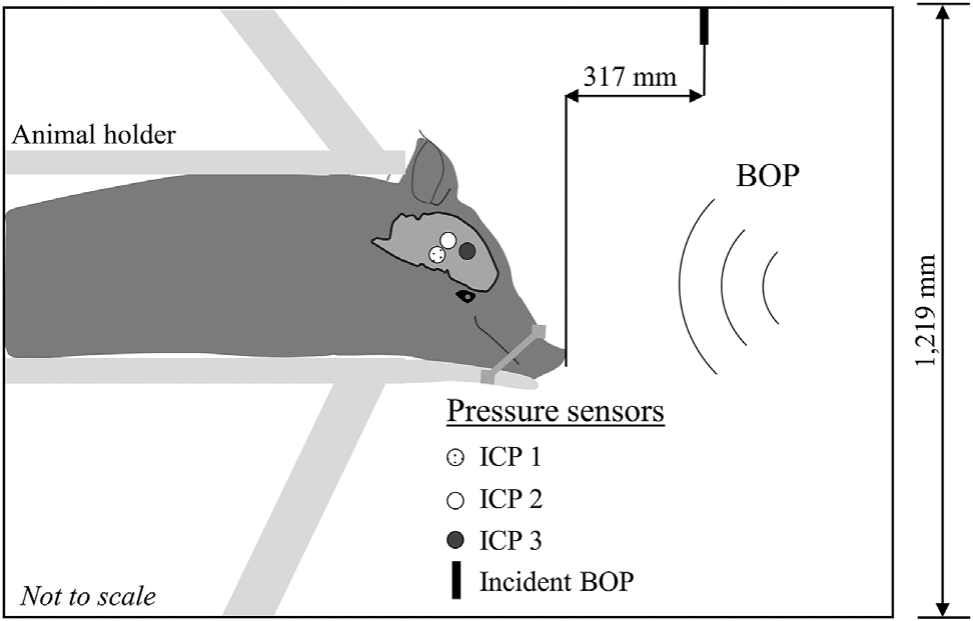
Schematic representation of the experimental setup. We conducted all the experiments in an Advanced Blast Simulator with a 1,219-mm square cross-section. We secured the minipig in a holder with the animal in the prone position and facing the incident blast overpressure (BOP). To minimize head motion during the experiments, we secured the snout to the holder with a cloth. We instrumented the animal with three pressure sensors implanted in the right hemisphere of the brain: the first sensor [Intracranial pressure 1 (ICP 1)] was deep and central, close to the mid-coronal plane; the second sensor (ICP 2) was superficially inserted in a ventricle near the mid-coronal plane; and the third sensor (ICP 3) was in the temporal lobe near the sphenoid. We measured the incident BOP 317 mm in front of the animal (Incident BOP).

First, we drilled a trephine through the margin of the squamous surface of the occipital bone at the transition to the nuchal crest, approximately 5 mm from the median plane. Next, we fitted a stainless steel thread insert into the trephine. Then, we used a customized cannula to hold the sensors and insert them through the trephine into the right hemisphere of the brain. We inserted the first sensor [intracranial pressure 1 (ICP 1)] deep and central, close to the mid-coronal plane; the second sensor superficially into a ventricle near the mid-coronal plane [intracranial pressure 2 (ICP 2)]; and the third sensor [intracranial pressure 3 (ICP 3)] in the temporal lobe near the sphenoid (Figure 1). Finally, after implanting the sensors, we removed the cannula and sealed the trephine using a drilled-cap screw with a hole in the center that acted as a conduit for the sensor wires. To achieve a watertight seal, we packed the conduit with dental acrylic. Before sealing the trephine, we nicked a small cerebral vein to evacuate air from the intracranial space.

#### Blast Testing

We exposed each minipig to a targeted incident BOP of 210 kPa twice in an advanced blast simulator (ABS) located at the Virginia Tech Center for Injury Biomechanics. The ABS is a combustion-based shock tube that uses a combination of oxygen and acetylene as fuel. Briefly, the ABS consists of driver (1,067 mm), transition (2,270 mm), test (1,829 mm), and end-wave eliminator (3,103 mm). The test section of the shock tube, where we placed the animals during the experiments, has a square cross-section with a side length of 1,219 mm.

To produce a blast wave, we separated the driver section from the rest of the shock tube using an acetate membrane, pumped a fuel mixture of oxygen and acetylene into the driver section, and ignited the mixture with an electric match. The combustion of the fuel mixture instantaneously increased the pressure in the driver section, and when the pressure reached a critical value, the acetate membrane broke and released the high-pressure combustion products into the transition section of the shock tube to generate a blast wave.

We secured the minipig in a prone position using a custom-built holder and placed it inside the test section of the shock tube facing the blast wave at a distance of 4,000 mm from the membrane. Because our goal was to investigate the biomechanical responses in the brain solely due to initial interaction of the blast wave with the head, we minimized the head motion by wrapping a cloth strap around the animal’s snout and the holder, which tilted the head downward. Using ImageJ 1.53h (National Institutes of Health, Bethesda, MD), we analyzed X-ray images of the animal in the shock tube and determined that the head of the animal was tilted downward at an angle of 43°. We recorded the temporal profile of the incident BOP produced by the blast wave at a distance of 317 mm from the front of the animal, using a pressure sensor (Model 8540-200; Endevco, Sunnyvale, CA) with its sensing element oriented parallel to the flow of the blast wave (Figure 1).

For the temporal profile of the incident BOP, we used a TMX Multi-Channel High-Speed Data Acquisition Recorder (AstroNova, Inc., West Warwick, RI) with piezoresistive bridge-interface cards to acquire data at a sampling frequency of 200,000 samples per second. For the intracranial pressure sensors, we used a TDAS system (Diversified Technical Systems, Inc., Seal Beach, CA) to acquire the data at a sampling frequency of 100,000 samples per second. The Virginia Tech (Blacksburg, VA) Institutional Animal Care and Use Committee (IACUC) as well as the Animal Care and Use Review Office (ACURO) of the U.S. Army Medical Research and Development Command (USAMRDC, Fort Detrick, MD) approved all experimental protocols related to the shock-tube experiments.

### Material Testing and Imaging

To develop the material model and geometry of a Göttingen minipig head, we performed material testing at low and high strain rates on brain and vasculature tissue samples and collected images of the different anatomical components of the head, such as the brain, dura, skull, soft tissue, and vasculature. Specifically, we performed shear tests on the brain samples and axial-tension tests on the vasculature samples to characterize their material behavior. To develop the geometry, we acquired CT images of the skull and surrounding soft tissue, MRI of the brain and dura, and µCT images of the vasculature. We conducted the material testing and imaging at the University of Utah Head Injury and Vessel Biomechanics Lab and the Preclinical Imaging Core Facility (Salt Lake City, UT), respectively, where the IACUC at the University of Utah as well as the ACURO of the USAMRDC approved all procedures for imaging and material testing.

#### Shear Tests on Brain Tissue

To characterize the Göttingen minipig brain-material behavior, we performed shear tests on three brain regions: *1*) cerebrum, *2*) cerebellum, and *3*) brainstem. To acquire the tissue samples, first, we sectioned the brain into thick coronal slices. Next, using a customized square die, we extracted tissue samples with a square cross-sectional length of 8 mm from all three regions. The thickness of the samples extracted from the cerebrum and cerebellum was 7 mm each, and the thickness of the sample from the brainstem was 5 mm. Then, we mounted the tissue samples between two parallel plates using cyanoacrylate adhesive. Finally, we performed shear tests by fixing the bottom plate and applying displacement loading on the top plate in the direction parallel to the plates.

To test the tissue behavior at a low strain rate, we applied a displacement of 8 mm at a strain rate of 0.01 s^−1^. We also performed high-strain-rate tests at 150.00 and 300.00 s^−1^ by applying displacement loading on the tissue samples until they failed. Because the brain tissue is not under loading *in vivo*, we did not precondition the tissue samples and applied the displacement loading immediately after mounting the tissue. We performed shear tests on six samples for each of the three brain regions and at the three strain rates (0.01, 150.00, and 300.00 s^−1^) and recorded the force-displacement data.

#### Axial-Tension Tests on Vasculature

To deduce the material properties for the vasculature, first, we resected middle-cerebral artery samples from the minipig brain, while ensuring to remove the surrounding brain tissues. Next, we removed the pia-arachnoid complex and ligated the branching arteries. Then, we mounted the artery onto the cannula of a customized testing device and secured it with a 6–0 suture. We applied cyanoacrylate adhesive on vessel tissue distal to the suture to hold it firmly during testing. In our previous work that characterized the middle cerebral artery from rats at high strain rates, we discussed in detail our device operations and testing procedures (Bell et al., 2018). Briefly, we preconditioned the samples by cycling the luminal pressure from 50 to 150 mmHg at five different levels of axial stretch from 1.0 to 1.1 times the *in vivo* length. Following preconditioning, we performed axial-tension tests to failure at three strain rates (0.01, 150.00, and 300.00 s^−1^) with the vessel sample at 80 or 120 mmHg luminal pressure, and measured the force and displacement data. Because the measured force and displacement data were similar between the samples tested at 80 and 120 mmHg luminal pressure, we combined these results when we assessed each of the three strain rates (*n* = 12 for each strain rate).

#### Imaging of Göttingen Minipig Head and Vasculature

To prepare the animal for scanning, first, we anesthetized the minipig with isoflurane and injected 12,000 units heparin to reduce thrombosis. Next, under anesthesia, we euthanized the minipig *via* exsanguination with a soft fixative Proflow (Dodge Company, Billerica, MA) and drained the blood from the vasculature by cutting the lateral saphenous vein and elevating the upper body. Then, we decapitated the animal and performed a CT scan under Vimago volumetric imaging (Epica Animal Health, San Clemente, CA) at 200-μm resolution. Following the whole-head CT imaging, we removed the soft and hard tissues surrounding the braincase, while preserving the braincase to maintain the integrity and shape of the brain. Finally, using a T-2 weighted protocol, we scanned the braincase under a Biospec 7T MRI (Bruker, Billerica, MA) at a resolution of 250 μm.

**FIGURE 2:**
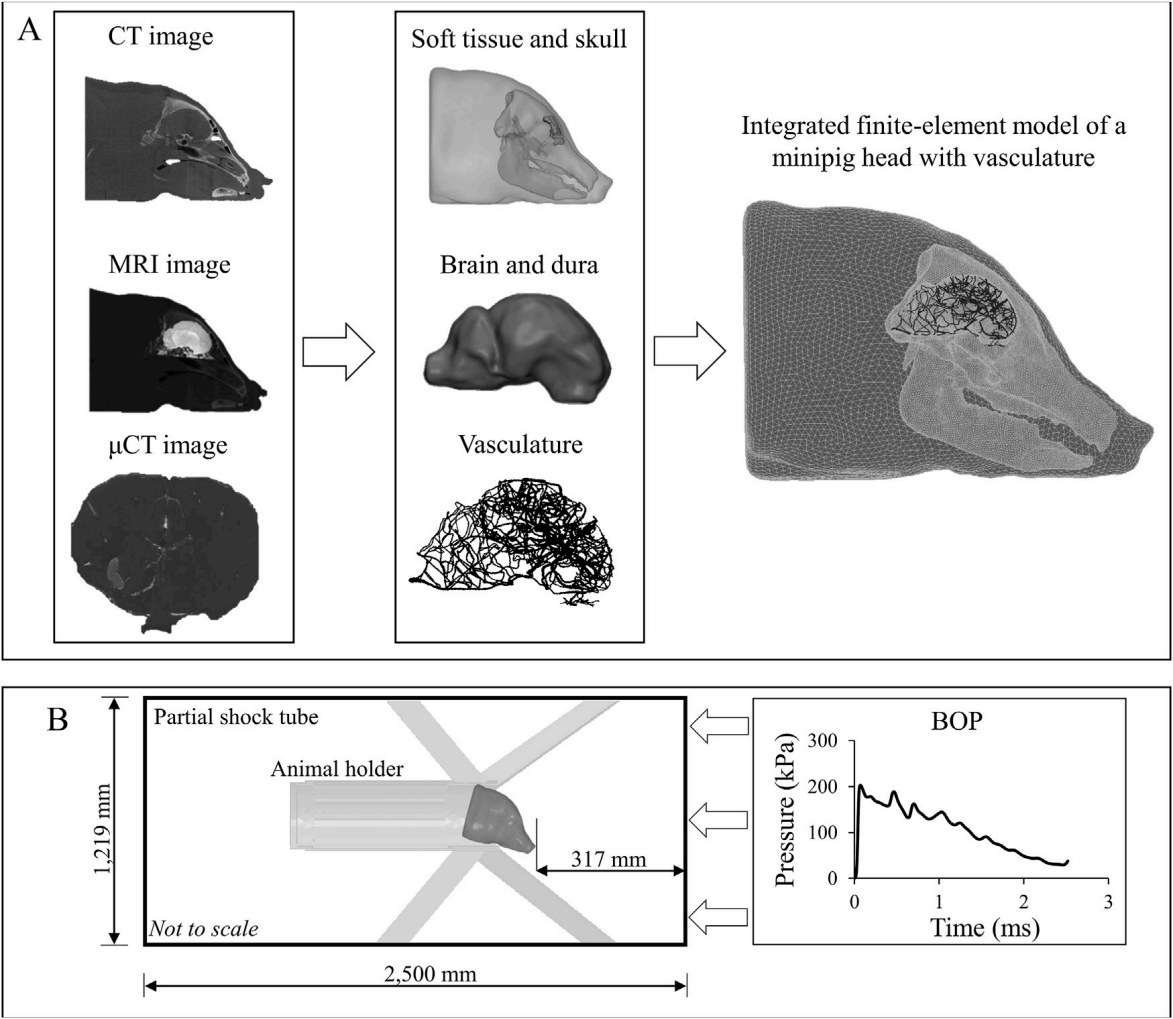
Development of a high-fidelity three-dimensional (3-D) finite-element (FE) model of a Göttingen minipig and the computer simulation setup. **(A)** To develop the high-fidelity 3-D FE model of a Göttingen minipig, first, we used computed tomography (CT) scans of the skull and surrounding soft tissue, magnetic resonance imaging (MRI) scans to capture the geometry of the brain and dura, and micro-computed tomography (μCT) scans to acquire the details of the vasculature. Next, using these scans, we developed two-dimensional surface models and converted them into 3-D FE models. Then, we integrated the FE models of the soft tissue, skull, dura, brain, and vasculature to create a FE model of a minipig head. **(B)** To perform the blast simulations, we combined the integrated minipig-head FE model with the FE model of the animal holder and coupled them with a 3-D partial shock-tube FE model. Using the experimentally measured incident blast overpressure (BOP) as input to the shock-tube model, we performed computer simulations with the minipig head in the prone position facing the BOP.

To acquire μCT images of the vasculature, we used the same methodology developed in our previous work for rats (Unnikrishnan et al., 2019). First, we perfused the brain (300 ml kg^−1^ of estimated braincase structure weight over a time of approximately 5 min) with BriteVu (Scarlet Imaging LLC, Murray, UT) at 55°C *via* the internal carotid artery. Then, we submerged the head into crushed ice to solidify the BriteVu. Finally, we performed high-resolution μCT imaging using a Siemens Inveon PET/CT scanner (Siemens Medical Solutions, Malvern, PA) at a resolution of 35 μm (Figure 2A). Because this perfusion process may denature the vascular tissue, it was only used for imaging purposes, and not for material testing.

### Development of a FE Model of the Göttingen Minipig

#### Geometries and FE Meshes

To develop a FE mesh, first, we imported the scanned images in DICOM (digital imaging and communications in medicine) format into the Mimics 19.0 software (Materialise, Leuven, Belgium). Next, we segmented the images using a semi-automated approach to create initial two-dimensional (2-D) surface geometries for the vasculature, brain, dura, skull with detailed sinus cavities, and soft tissue around the skull. Then, we exported these 2-D geometries into 3-Matic 11.0 (Materialise, Leuven, Belgium) and manually improved their quality by removing discontinuities and smoothing sharp angles (Figure 2A). Finally, we imported the improved 2-D surface geometries into Hypermesh 2017.1 (Altair Engineering, Troy, MI) and converted them into a 3-D volume mesh. For the brain, dura, skull, and soft tissue, we used tetrahedral (4-noded) solid elements for meshing, while we used linear triangular (3-noded) elements to mesh the vasculature.

We imported the 3-D volume mesh models into Abaqus 2017 (Dassault Systèmes, Vélizy-Villacoublay, France) and converted the linear elements of the brain, dura, skull, and soft tissue into quadratic tetrahedral (10-noded) solid elements (C3D10M). This quadratic element minimizes the volumetric locking that may arise when modeling nearly incompressible materials, such as the brain tissue (Fernandes et al., 2018). We retained the linear triangular elements for the vasculature and assigned element type S3 and a shell thickness of 0.054 mm. We assigned the shell thickness based on our measurement of Göttingen minipig middle-cerebral artery samples (0.054 ± 0.010 mm). Using the merge tool in Abaqus, we combined the brain, dura, skull, and soft tissue into one seamless solid. Using Abaqus’s embedded-elements technique, we coupled the vasculature and the head volume mesh to develop an integrated FE model of a minipig head with the vasculature (Figure 2A) (Unnikrishnan et al., 2019; Unnikrishnan et al., 2021).

#### Material Properties

We considered the brain (cerebrum, cerebellum, and brainstem), vasculature, soft tissue, and dura as incompressible materials with a bulk modulus of 2.19 GPa. Based on previous compression test data (540.00 s^−1^) from pig muscle tissue samples (Song et al., 2007), we modeled the deviatoric response of the soft tissue as a hyperelastic material using a two-term Ogden model. Using uniaxial-tension test data (0.01 s^−1^) from pig dura-arachnoid mater samples (Pierrat et al., 2020), we modeled the deviatoric response of the dura as a hyperelastic model using a one-term Ogden model. We defined the skull as a compressible, linear-elastic material with an instantaneous elastic modulus of 2.00 GPa and a Poisson’s ratio of 0.22 (Alexander et al., 2019). We modeled the deviatoric response of the brain tissue and vasculature as hyperelastic, linear-viscoelastic materials using a one-term Ogden model with a one-term Prony series based on the experiments performed on the brain tissue and vasculature (*Shear Tests on Brain Tissue* Section and *Axial-Tension Tests on Vasculature* Section). Table 1 summarizes the material properties for the different anatomical components of the Göttingen minipig-head FE model.

**TABLE 1:** Summary of the material properties used for the individual anatomical components included in the high-fidelity minipig-head model.

Components	Density (*kg m* ^*−3*^)	Elastic constants		Hyperelastic constants					Viscous constants	
		*Elastic modulus (GPa)*	*Poisson´s ratio*	*Bulk modulus (GPa)*	*Shear modulus (kPa)*		*Material constant*		*g*	*Decay constant* (*s* ^*−1*^)
					*µ*_*1*_	*µ*_*2*_	*α*_*1*_	*α*_*2*_		
Skull	1,200	2.00	0.22	2.19	0.35	38.5	23.4	-5.3
Soft tissue	1,040							
Dura	1,040			2.19	450.00		16.5	
Cerebrum	1,040			2.19	1.81		10.1		0.99	0.276
Cerebellum	1,040			2.19	1.46		8.0		0.92	0.562
Brainstem	1,040			2.19	2.25		11.2		0.99	0.455
Vasculature	1,040			2.19	700.40		12.6		0.90	0.077

#### FE Model of the Shock Tube and Blast Simulations

We developed a 3-D FE model of a partial shock tube to represent the ABS used in the experiments. The shock-tube model had a square cross-section with a side length of 1,219 mm and an overall length of 2,500 mm (Figure 2B). We modeled the air in the shock tube as an ideal gas (density of 1.23 kg m^−3^ and specific gas constant of 287 J kg^−1^ K^−1^) at a temperature of 300 K and assigned linear hexahedral (8-noded) Eulerian elements (EC3D8R). To simulate blast loading in the model, we applied the experimentally measured temporal profile of the incident BOP as a pressure boundary condition at the inlet of the shock-tube model (Figure 2B). Moreover, on the sides and the outlet of the shock tube, we constrained the velocity of the air perpendicular to the walls to zero.

To perform blast simulations, we combined the minipig-head model with the holder FE mesh (modeled as a discretely rigid shell) and coupled them into our partial shock-tube model using the coupled Eulerian-Lagrangian approach in Abaqus. To mimic the experimental setup, we tilted the head model downward at an angle of 43°, fixed the holder, and constrained the linear displacements, i.e., the motion in x, y, and z directions, on the nodes on the underside of the snout of the minipig-head model. We performed all simulations using Abaqus/Explicit on a SGI 8600 system termed Mustang at the U.S. Air Force Research Laboratory Supercomputing Resource Center. Using 48 CPU cores and a stable time step of 28 ns determined by the double-precision Abaqus solver, we completed 5-ms simulations in 35 h. We post-processed the simulation results using the CAE module in Abaqus 2017.

Using our model, we first investigated how the blast wave evolves and loads the brain. Next, we assessed the biomechanical responses, such as pressure and maximum principal strain, at three different locations in the brain. Finally, we determined the contribution of the cerebral vasculature on the biomechanical responses by comparing the responses obtained using minipig-head models with and without the vasculature.

## Results

### Experimental Pressure Measurements

We performed blast-exposure experiments on five animals, with each animal exposed to a BOP of 210 kPa twice, and recorded the intracranial pressure at three locations in the brain. We determined the target value of 210 kPa based on pilot tests, wherein we observed a 70% survival rate when we exposed animals to this BOP. We reviewed the entire dataset, i.e., 30 temporal profiles of pressure, and discarded all 10 recordings for ICP 3 because its temporal profile contained sharp negative or positive spikes. After analyzing the measurements for ICP 1 and ICP 2, we identified similar pressure spikes in the temporal profile for five and six recordings, respectively, and discarded them. In addition, we also discarded one recording for ICP 1 due to a sensor malfunction. For our analyses, we used four pressure measurements each for ICP 1 and ICP 2 from six different blast exposures.

**FIGURE 3:**
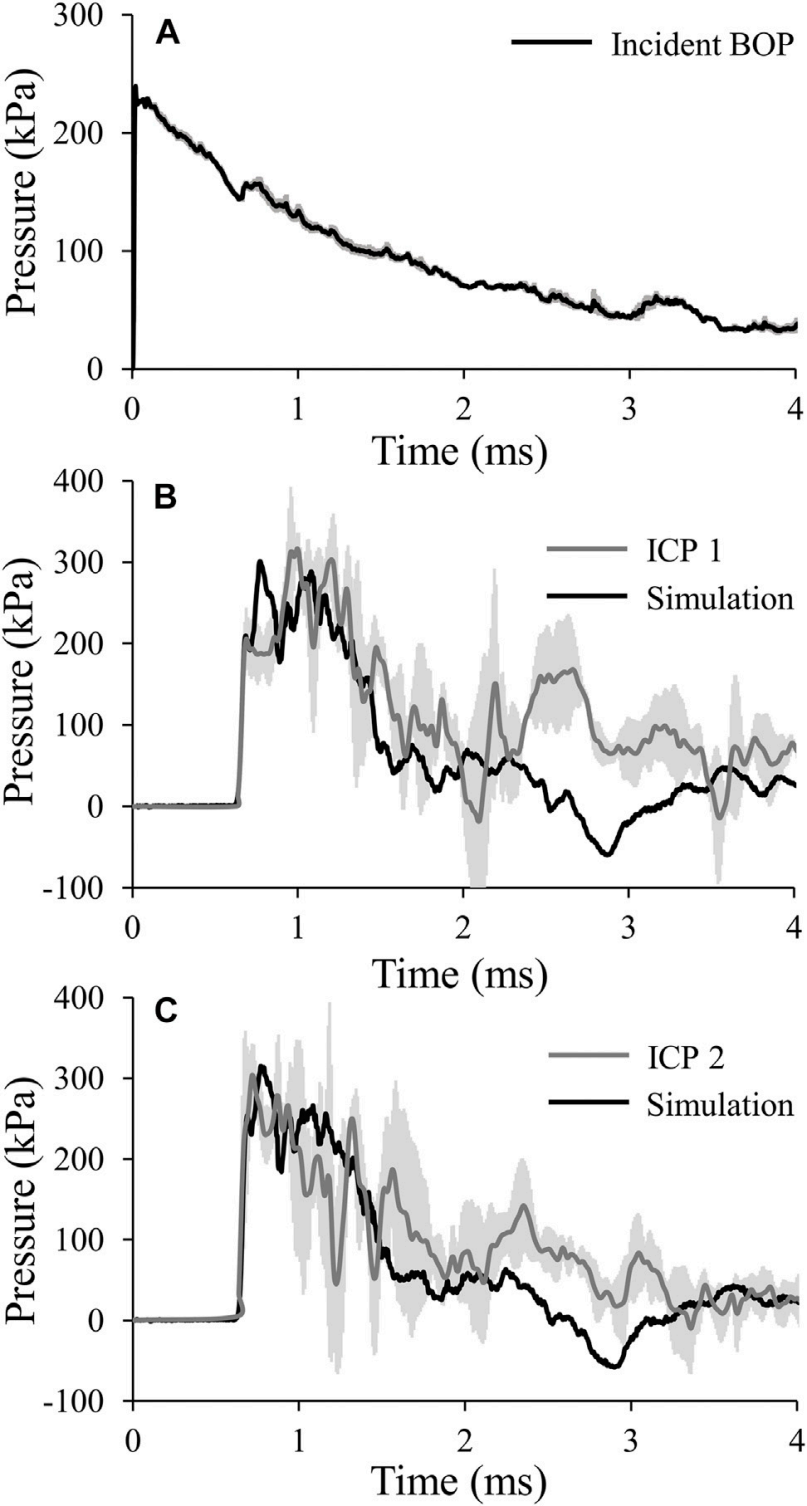
**(A)** Temporal profile of the incident blast overpressure (BOP). The solid line and shaded area represents the mean (*n* = 6) and two standard errors of the mean (SEM), respectively. (Because the SEM are small, they may not be visible.) **(B)** and **(C)** Comparison of the pressures predicted by the finite-element model of the minipig head with vasculature (black lines) with those obtained from the experimental studies (gray lines) for intracranial pressure 1 (ICP 1; B) and intracranial pressure 2 (ICP 2; C). The gray lines and shaded areas represent the mean (n = 4) and two SEM, respectively.

For the targeted incident BOP of 210 kPa, the temporal profile showed an instantaneous rise to maximum pressure followed by a nearly monotonic decay (Figure 3A), with a measured maximum incident pressure of 238.70 ± 6.22 kPa [mean ± two standard errors of the mean (SEM)]. In contrast, the average intracranial pressure measured at ICP 1 increased to its maximum value in two steps: first, it increased instantaneously to 205.81 ± 38.37 kPa (at time t = 0.69 ms), remained relatively steady for about 0.18 ms, and then increased again at a slower rate to a maximum pressure of 316.95 ± 19.26 kPa (t = 0.99 ms) (Figure 3B). After achieving the peak value, the pressure oscillated and formed a second peak at 1.20 ms. Similarly, at ICP 2, the average pressure initially increased instantaneously to 247.88 ± 91.82 kPa (t = 0.69 ms), then it further increased at a slower rate and reached a peak value of 303.16 ± 39.39 kPa at t = 0.73 ms (Figure 3C). Although we observed a decaying trend in the temporal pressure profiles at both locations, we also noted an oscillatory behavior after 1.35 ms.

### Constitutive Models for Brain Tissue and Vasculature

**FIGURE 4:**
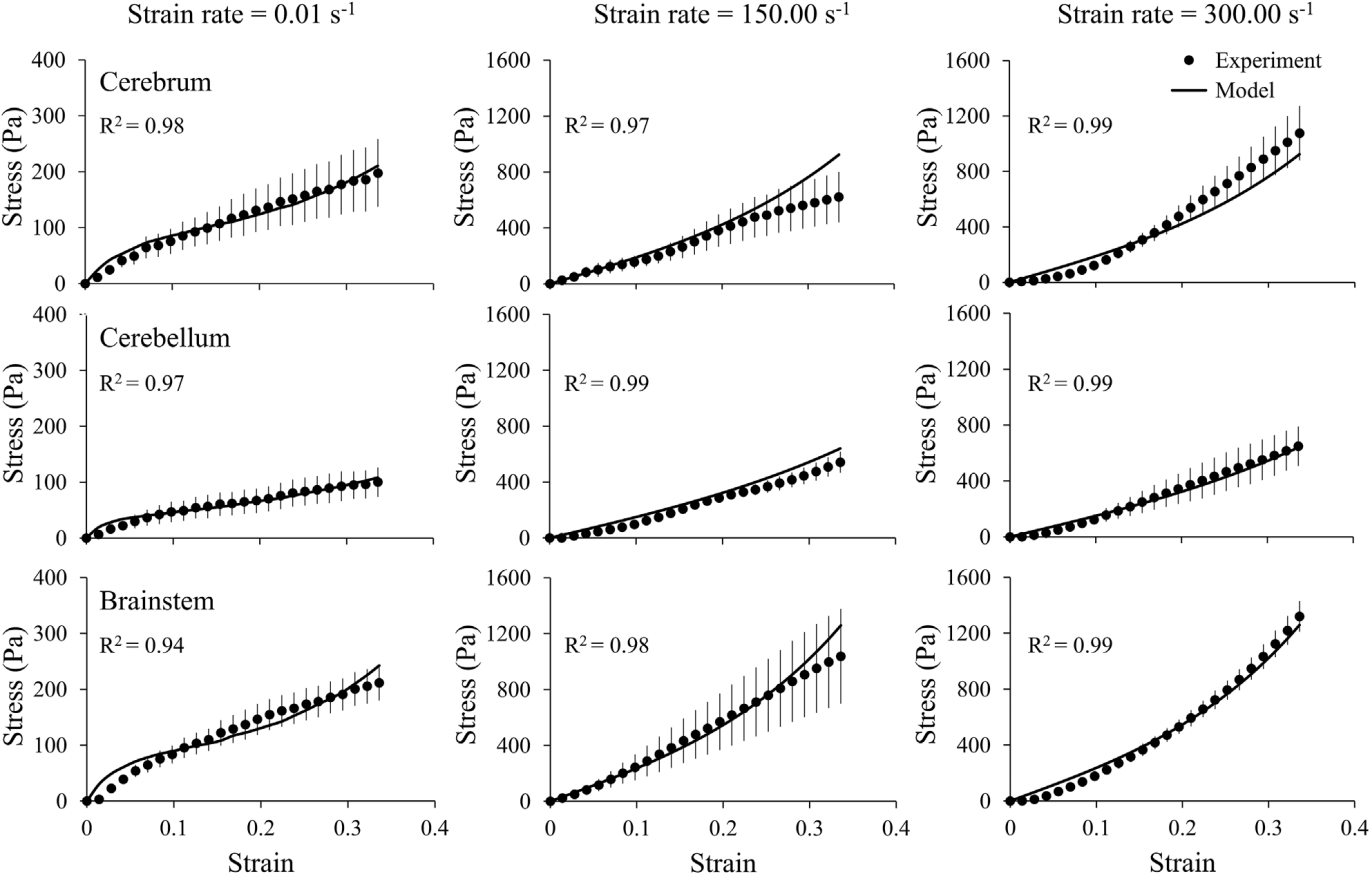
A one-term Ogden hyperelastic, one-term Prony series model (solid lines) fit to the average experimental stress-strain behavior (circles) for three brain regions (cerebrum, cerebellum, and brainstem), each at three strain rates (0.01, 150.00, and 300.00 s^-1^). The circles and vertical lines represent the mean (*n* = 6) and one standard error of the mean, respectively.

**FIGURE 5:**
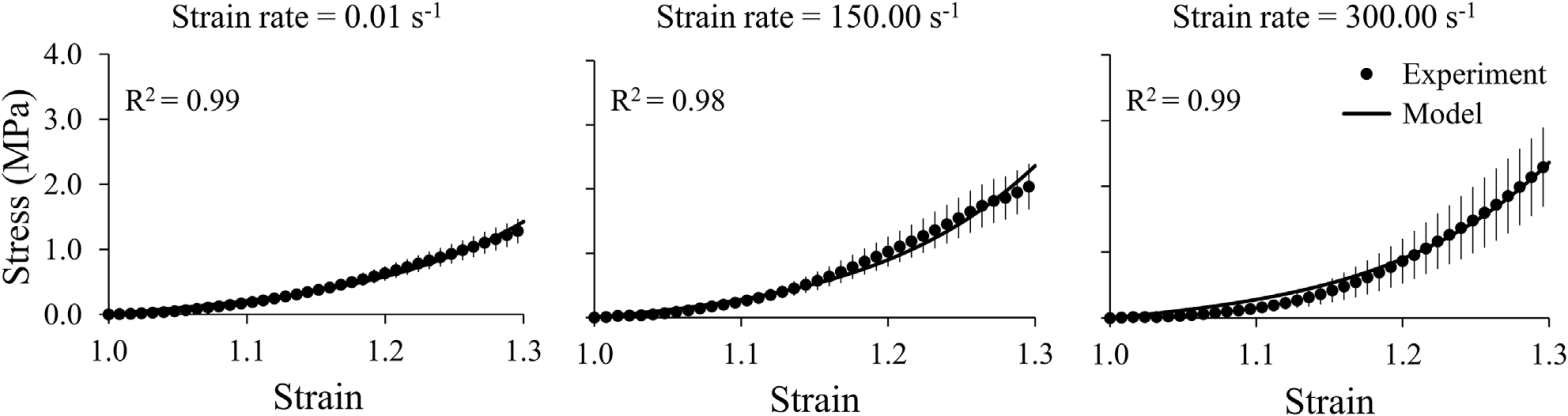
A one-term Ogden hyperelastic, one-term Prony series model (solid lines) fit to the average experimental stress-strain behavior (circles) for the middle cerebral artery samples at three strain rates (0.01, 150.00, and 300.00 s^-1^). The circles and vertical lines represent the mean (*n* = 12) and one standard error of the mean, respectively.

We obtained the stress and strain curves for the three brain regions (cerebrum, cerebellum, and brainstem) and the middle cerebral artery using the measured force, displacement, and sample dimensions at three strain rates, 0.01, 150.00, and 300.00 s^−1^ (Figures 4, 5). To accurately capture the nonlinear-elastic response and the loading-rate effects of both the brain and the vasculature, we assumed that they behaved as hyperelastic, linear-viscoelastic materials and derived the material properties by simultaneously fitting a one-term Ogden, one-term Prony series for the three loading rates (Table 1) (Unnikrishnan et al., 2019). We observed that our model fit the average experimental stress-strain behavior well (R^2^ > 0.94) in all cases (Figures 4, 5).

### Mesh-Sensitivity Tests and Model Validation

We performed mesh-refinement studies on the shock-tube and minipig-brain FE models using three mesh configurations for each model (Table 2). For both models, we systematically increased the number of elements in the mesh and evaluated the maximum pressure near the nose in the shock-tube model and at the center of the mid-sagittal plane in the brain model. Specifically, for the shock-tube model, the maximum pressure predicted by the current model with 852,550 elements (T3 in Table 2) was less than 1.0% different from the maximum pressure predicted by a model with 717,715 elements (T2 in Table 2). Conversely, we observed a pressure difference of 5.1% between a model with 613,312 elements (T1 in Table 2) and T2, indicating that further mesh refinement is not necessary for T3. For the brain model, we observed a difference of only 2.5% in the maximum pressure between the current model with 305,352 elements (B3 in Table 2) and a model with 209,503 elements (B2 in Table 2). However, when we compared a model with 67,879 brain elements (B1 in Table 2) and B2, the difference was 6.4%, indicating that further mesh refinement is not necessary for B3.

**TABLE 2:** Summary of the mesh-sensitivity tests performed on the shock-tube model and the minipig-brain model.

Model	Number of elements	Element size (mm)	Maximum pressure (kPa)
Shock-tube model			
T1	613,312	9.5	264
T2	717,715	8.5	278
T3^a^	852,550	7.5	279
Brain model			
B1	67,879	2.0	420
B2	209,503	1.5	448
B3^a^	305,352	1.0	459

a Selected.

To validate our model, we compared the experimentally measured temporal profiles of the pressure at the two sensor locations in the brain with the minipig-head model predictions for the incident BOP of 210 kPa (Figure 3). We noted that our model also predicted the two-step pressure increase seen in the experiments. Specifically, for ICP 1, the pressure initially increased instantaneously to 209.83 kPa (at time t = 0.69 ms), followed by a further increase at a slower rate to a peak value of 300.76 kPa (t = 0.78 ms), a difference of 6% compared to the experimentally measured maximum pressure. Although the model predicted the two-step pressure increase observed in the experiments, we noted a phase-shift difference between the measured and predicted maximum pressures, with the predicted value preceding the experimental one by 0.21 ms. Next, as observed in the experiments, the pressure oscillated and formed a second peak at time t = 1.08 ms. For ICP 2, initially our model predicted the sharp pressure rise to 254.32 kPa (t = 0.69 ms), followed by a further pressure increase at a slower rate to a maximum value of 315.15 kPa (t = 0.77 ms), a difference of 4% compared to the experimentally measured maximum pressure. In this case, we observed a minor phase-shift difference between the measured and predicted maximum pressures, with the experimentally measured peak preceding the model predicted one by 0.04 ms. Although, our model closely matched the decaying trend observed in the measurements of ICP 1 and ICP 2, it did not predict the oscillations in the temporal pressure profiles after 1.35 ms.

### Blast Overpressure Evolution and Loading

**FIGURE 6:**
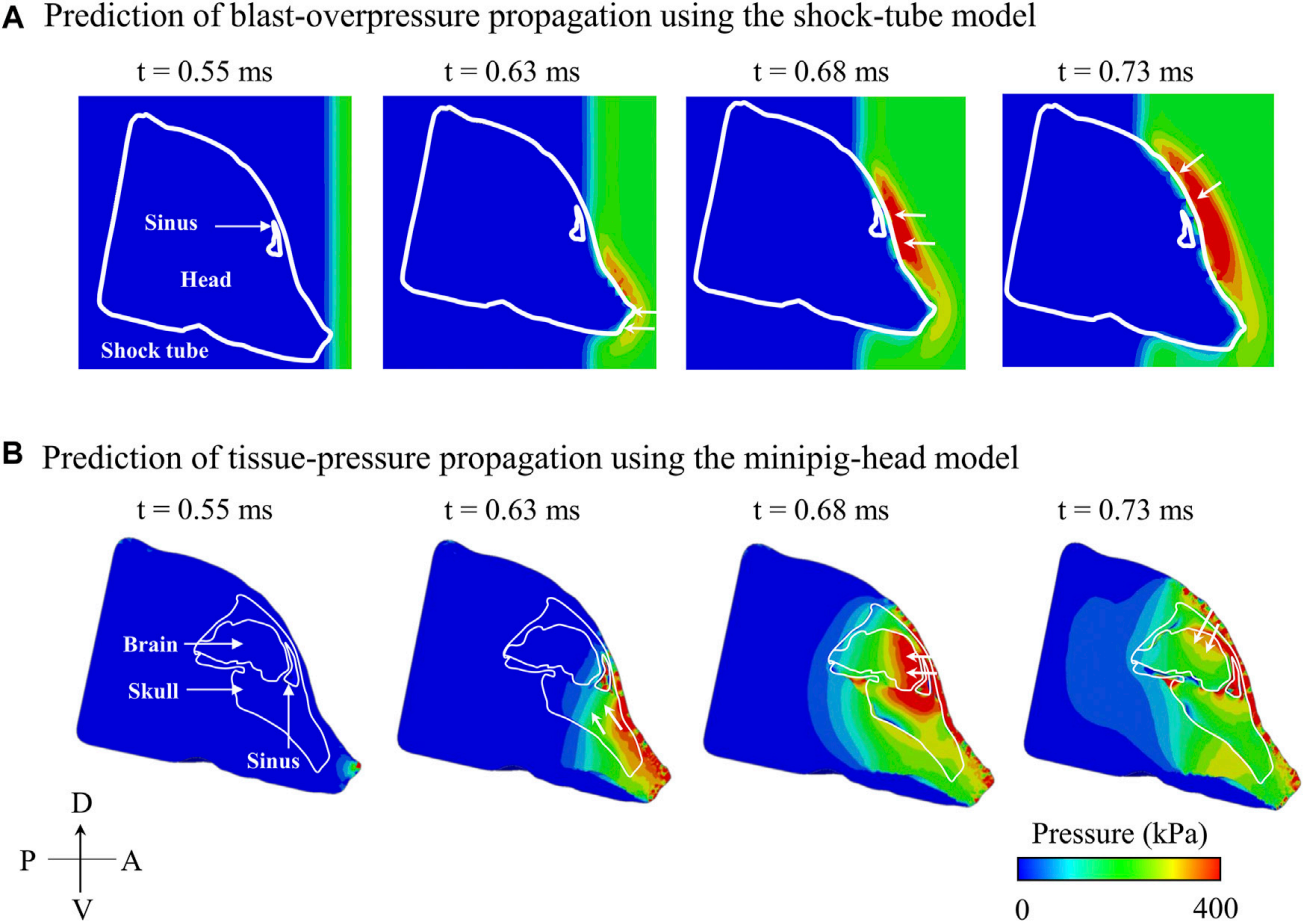
Predicted temporal and spatial propagation of blast overpressure (BOP) and tissue pressure based on the **(A)** shock-tube model and **(B)** minipig-head model, respectively, along the mid-sagittal plane. **(A)** Based on the shock-tube model, at time t = 0.55 ms, the BOP reached and loaded the head. At time t = 0.63 ms, the BOP loaded the snout (white arrow); at time t = 0.68 ms, the BOP traversed and loaded the forehead near the sinus (white arrow); and at time t = 0.73 ms, the BOP loaded the top of the head (white arrow). **(B)** Based on the minipig-head model, at time t = 0.55 ms, the pressure started to load the brain tissues. At time t = 0.63 ms, the pressure in the snout traveled through the skull and loaded the brain (white arrows); at time t = 0.68 ms, the pressure from the forehead entered the sinus cavity and loaded the brain (white arrows); and at time t = 0.73 ms, the pressure from the top of the head entered through the skull and loaded the brain (white arrows). A: anterior; D: dorsal; P: posterior; V: ventral.

Using the coupled FE model of the shock tube and the minipig head, we assessed the evolution of pressure in the shock tube (Figure 6A) and tissues (i.e., the soft tissue, skull, and brain) of the minipig head (Figure 6B). First, at time t = 0.55 ms, the BOP interacted with the tip of the nose initiating a pressure wave in the tissues. Next, at time t = 0.63 ms, the pressure wave in the tissues propagated and loaded the brain, while the BOP loaded the snout. Then, at t = 0.68 ms, the BOP loaded the forehead of the minipig near the sinus, while a corresponding pressure wave loaded the anterior region of the brain. Finally, at time = 0.73 ms, the BOP loaded the top of the head and, simultaneously, we observed a corresponding pressure wave loading the dorsal region of the brain.

### Effect of Vasculature on the Biomechanical Responses

**FIGURE 7:**
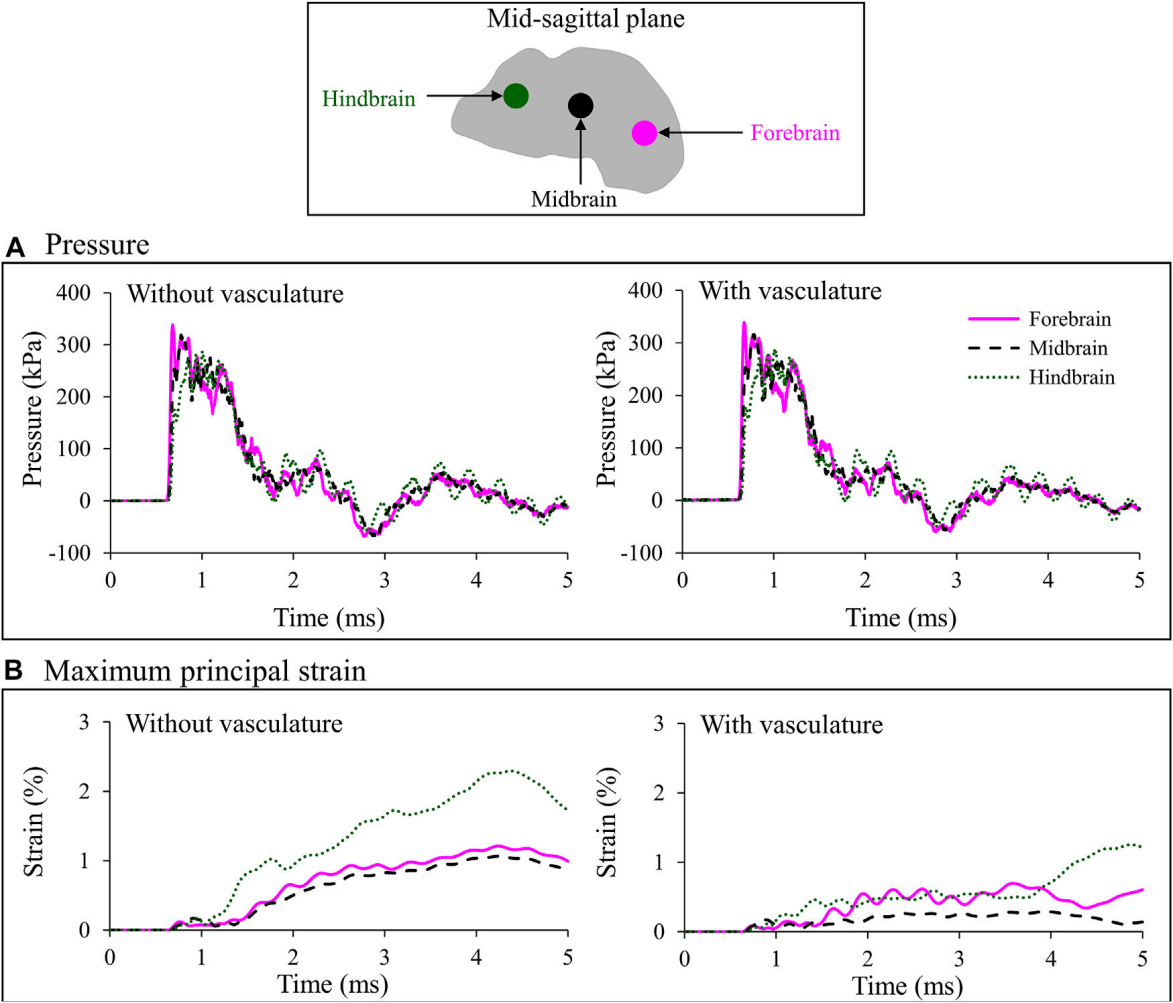
Comparison of the temporal evolution of **(A)** pressure and **(B)** maximum principal strain predicted by the minipig-head model with and without vasculature at three locations (i.e., forebrain, midbrain, and hindbrain) along the mid-sagittal plane. Locations are indicated by colored circles on the mid-sagittal plane of the brain. The similarity in the pressure profile for the model results with and without vasculature indicates that inclusion of the vasculature did not influence the evolution of pressure in the brain tissue **(A)**. In addition, during the initial 1 ms, we observed similar strain predictions for the two models because until that time the blast wave had not yet loaded the head **(B)**. However, once that happened, we observed a reduction in brain-tissue strain caused by the stiffening of the brain tissue in the model with vasculature.

To quantify the effect of the vasculature, we assessed the temporal profiles of the pressure and maximum principal strain close to the interface between the vasculature and the brain tissue, at three locations (forebrain, midbrain, and hindbrain) along the mid-sagittal plane (Figure 7). Based on models with and without vasculature, the maximum pressure peaked at the forebrain and decreased by approximately 6 and 15% at the midbrain and hindbrain, respectively. As expected, at all three locations, the maximum pressure and temporal profiles of the pressure were nearly identical for both models (Figure 7A).

Without vasculature, the maximum principal strain increased slowly in the brain and reached a peak value of 1.2% in the forebrain, 1.1% in the midbrain, and 2.3% in the hindbrain (Figure 7B, left panel). In contrast, the pressure increased instantaneously and decayed with a somewhat monotonic trend with time (Figure 7A, left panel), similar to that of the BOP (Figure 3A). The peak strains at the midbrain and forebrain were, respectively, 54 and 47% lower than the peak value at the hindbrain (Figure 7B, left panel). With vasculature, the maximum principal strain reached a peak value of 0.7% in the forebrain, 0.3% in the midbrain, and 1.2% in hindbrain (Figure 7B, right panel). Comparison of maximum principal strain with and without vasculature at the three locations in the brain revealed that the strains were lower with the vasculature, as expected. Specifically, the peak values of the predicted strains were approximately 42, 73, and 45% lower with vasculature compared to those without vasculature at the forebrain, midbrain, and hindbrain, respectively (Figure 7B). It is important to note that these comparisons are for the first 5 ms of the blast exposure, only considering the initial interaction of the blast wave with the head.

### Spatial and Temporal Evolution of Maximum Principal Strain With and Without Vasculature

**FIGURE 8:**
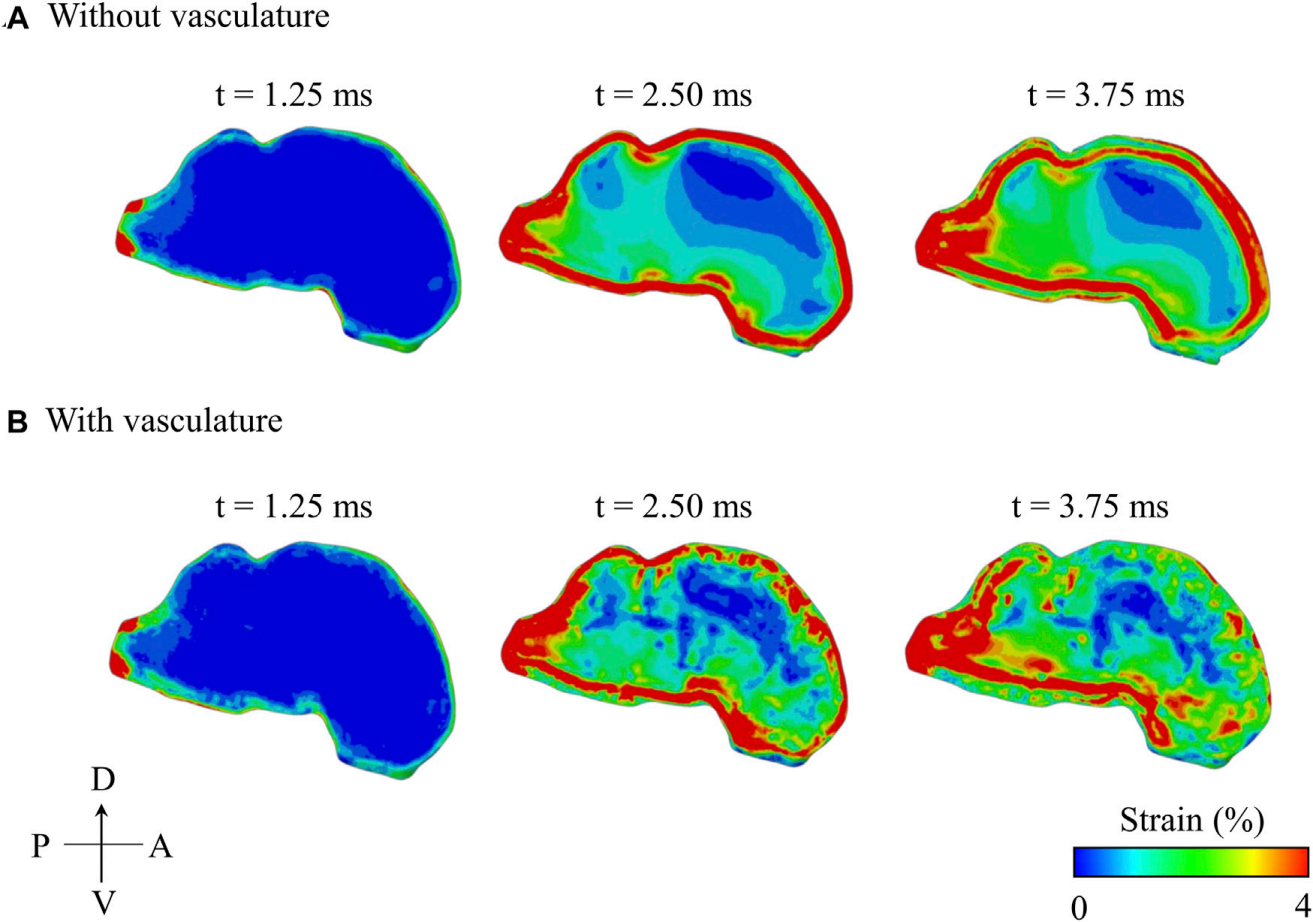
Comparison of the predicted temporal and spatial propagation of maximum principal strain along the mid-sagittal plane of the minipig head **(A)** without vasculature and **(B)** with vasculature. A: anterior; D: dorsal; P: posterior; V: ventral.

The maximum principal strain started at the peripheral regions of the brain and propagated inwards towards the center of the brain with time (Figure 8). In contrast, the pressure wave traveled from anterior to posterior in the brain along the direction of the BOP propagation (i.e., from right to left in Figure 6B). With and without vasculature, at all three time points (t = 1.25, 2.50, and 3.75 ms), we observed large strain values in the posterior region of the brain (i.e., the brainstem). Without vasculature, the strain was largely concentrated at the peripheral regions of the brain. However, with vasculature at t >1.25 ms, we observed large strain distributions with pockets of elevated strains in the interior of the brain, especially at t = 3.75 ms. Interestingly, as time progressed (i.e., from t = 2.50–3.75 ms), in the model with vasculature, we observed a reduction in the overall strain magnitude near the periphery of the dorsal and anterior-dorsal brain regions. Note that our results of the evolution of the maximum principal strain in the brain with and without vasculature are limited to the first 3.75 ms of the blast-wave propagation.

## Discussion

We developed a high-fidelity 3-D FE model of a Göttingen minipig head to characterize the biomechanical responses in the brain tissue resulting from a blast exposure in a laboratory shock tube. First, we collected MRI scans of a minipig head to capture the details of the brain and dura and utilized CT to acquire the details of the skull and surrounding soft tissue. Using the MRI and CT scans, we constructed the geometry and created a FE mesh of the minipig head. Next, we acquired µCT scans of the vasculature, used them to construct the geometry of a 3-D vasculature network, and integrated it into the FE mesh of the minipig head. Then, to establish material properties of the brain tissues and cerebral vasculature of Göttingen minipigs, we performed shear tests and axial-tension tests, respectively, at low and high strain rates. To account for regional variations in the material properties of the brain, we tested tissue samples from three locations: the cerebrum, cerebellum, and brainstem. Using these data, we computed hyperelastic, linear-viscoelastic material properties for the brain tissue and cerebral vasculature and incorporated them into our model. To validate the model, we performed blast-exposure experiments on Göttingen minipigs at an incident BOP of 210 kPa and compared the measured pressures at two locations in the brain tissue with the model predictions. Finally, using the validated FE model, we examined how the BOP interacted with the head to load the brain and investigated the influence of the vasculature on the predicted biomechanical responses in the brain.

The experimentally measured incident BOP exhibited an immediate rise to the maximum pressure followed by a smooth decay to the atmospheric pressure or baseline condition (Figure 3A). Conversely, ICP measured at the two locations in the brain increased to the maximum pressure in two steps: first, the pressure increased instantaneously to an intermediate value (at time t = 0.69 ms for both ICPs), followed by another rise at a slower rate to the maximum pressure (t = 0.99 ms for ICP 1 and t = 0.73 ms for ICP 2) (Figures 3B,C). Other blast-exposure studies on pigs have also observed similar trends in ICP measurements. For instance, Feng et al. (2016) performed open-field blast exposures on minipigs using a range of incident BOPs between 150 and 430 kPa and showed that the measured ICP reached its maximum value in two steps (Feng et al., 2016).

We validated our high-fidelity FE model of the minipig head by comparing the model-predicted temporal pressure profiles against the experimental measurements for ICP 1 and ICP 2 (Figures 3B,C). Our model predicted the two-step rise to maximum pressure for both ICP 1 (at time t = 0.69 and 0.78 ms) and ICP 2 (t = 0.69 and 0.77 ms). However, we observed phase-shift discrepancies of 24 and 5% for ICP 1 and ICP 2, respectively, when compared to the experimental results. We could not compare these discrepancies with other studies because such differences have not been previously reported (Zhu et al., 2013b; Kalra et al., 2017). The model also predicted the formation of the second peak (t = 1.08 ms) observed in the experiments for ICP 1. Zhu et al. (2013b) noted a similar behavior in their ICP predictions and experimental measurements and attributed this second peak to pressure wave reflections from the skull (Zhu et al., 2013b). Overall, we observed a good agreement between the model predictions and the experimental measurements, with discrepancies in the maximum pressure of less than 6% at both locations. Previous model validations have reported comparable or larger discrepancies. For example, Zhu et al. (2013b) observed a discrepancy of no more than 12% for the maximum pressure predictions in the brain for a Yorkshire pig model, while Kalra et al. (2017) reported differences between 3 and 60% at different brain locations for a Yucatan minipig model (Zhu et al., 2013b; Kalra et al., 2017). Finally, for both ICPs, our model was not able to predict the experimentally observed oscillations after 1.35 ms, which we believe resulted from vibrations of the animal holder during the experiments.

To gain insight into the cause of the two-step pressure rise observed for both ICP 1 and ICP 2, we used the model to determine how the incident BOP loaded the brain and analyzed the pressure propagation near the skull-brain interface on the anterior side of brain and in the air close to the head. At time t = 0.63 ms, we observed that the pressure wave generated in the snout propagated through the tissues and loaded the brain, while the BOP in the air was only loading the snout (Figure 6), confirming the well-established difference in the velocity of wave propagation in the tissue and the air (Ganpule et al., 2013). Indeed, we found that the pressure wave in the tissues propagated twice as fast as the BOP in the air. While the wave propagation velocity in the air between the tip of the nose and the forehead was 570 m s^−1^, the velocity in the tissues between the tip of the nose and the anterior region of the brain was 1,160 m s^−1^. This lag in the BOP air velocity led to three pressure-loading mechanisms in the brain. First, the pressure wave generated in the nasal region and snout propagated through the tissues and loaded the brain (t = 0.63 ms). Second, while the pressure from the first mechanism was still loading the brain, the pressure wave on the forehead entered through the skull, loaded the anterior region of the brain, and added to the existing tissue pressure (t = 0.68 ms) (Figure 6). This combination of loading from mechanisms 1 and 2 manifested itself as the first of the two-step pressure increases observed in ICP 1 and ICP 2 (at time t = 0.69 ms). In the third mechanism, the pressure wave from the top of the head propagated to the brain and combined with the existing tissue pressure (t = 0.73 ms), initiating the second step of the pressure increase at the mid-coronal plane at t = 0.78 and 0.77 ms for ICP 1 and ICP 2, respectively in Figure 3.

To assess the influence of the vasculature on the biomechanical responses, we compared the predicted pressures and maximum principal strains in the brain at three locations (forebrain, midbrain, and hindbrain), using models with and without vasculature (Figure 7). At each of the three locations, we predicted the responses on brain elements near the interface between the vasculature wall and the brain tissue for the with-vasculature case and chose elements at the identical positions for the without-vasculature case. Without vasculature, the pressure propagated from the anterior to the posterior end of the brain with the highest and lowest pressures observed at the forebrain and hindbrain, respectively (Figure 7A). Kalra et al. (2017) observed a similar reduction in maximum pressure propagating from the anterior to the posterior end of the brain in their minipig-head model (Kalra et al., 2017). For the model with vasculature, we used an identical bulk modulus of 2.19 GPa for the brain and the vasculature, while employing significantly stiffer shear properties for the vasculature (Table 1). As expected (Unnikrishnan et al., 2019), when compared to the results of the without-vasculature model, we did not observe a substantial difference in either the maximum pressure or pressure profile at all three locations (Figure 7A). The similarity of the predictions indicates that the brain pressure depends on the bulk modulus and that the shear properties of the brain contribute little to the pressure predictions.

Without vasculature, the maximum principal strains at all three locations increased gradually and reached their maximum values at approximately the same time (4.30 ms). Our predictions showed that the maximum principal strain was considerably higher in the hindbrain than in the forebrain and midbrain (Figure 7B, left panel). To determine the cause of the high strain in the hindbrain, we evaluated the spatial propagation of the maximum principal strain and observed high strain in the posterior region of the brainstem, i.e., near the interface between the foramen magnum and the brainstem, at time t = 1.25 ms (Figure 8A). As time progressed, the strain propagated inward and increased throughout the brainstem. Zhu et al. (2013b) made similar observations of high strain in the brainstem compared to other locations in the brain using their pig model (Zhu et al., 2013b). They proposed that this difference in the strain between the brainstem and other brain regions resulted from the skull walls at the foramen magnum pressing on and deforming the brainstem tissue.

With vasculature, the maximum principal strain decreased by 42, 73, and 45% at the forebrain, midbrain, and hindbrain, respectively (Figure 7B). This expected reduction in strain was due to the increase in the stiffness of the brain tissue caused by the inclusion of the vasculature. We observed similar results in our previous study using a high-fidelity FE model of a rat head, where we predicted a reduction in the brain-tissue strains after embedding vasculature in the model (Unnikrishnan et al., 2019). Similarly, using a surrogate human-head model, Hua et al. (2015) described that the vasculature did not influence the predicted pressures but significantly altered the strains during blast exposures (Hua et al., 2015).

To further delineate the influence of the vasculature, we compared the spatial propagation of the maximum principal strain along the mid-sagittal plane with and without vasculature as a function of time (Figure 8). Without vasculature, we observed a high strain in the periphery of the brain (t = 2.50 ms), with the strain propagating towards the center over time. When compared to the model without vasculature, with vasculature, strain decreased near the periphery of the dorsal and anterior-dorsal regions of the brain between time t = 2.50 ms and t = 3.75 ms due to the stiffening of the brain tissue. Interestingly, when we compared the strain distribution predictions with and without vasculature at time t = 3.75 ms, we observed a larger strain distribution with pockets of high strain in the interior of the brain for the with-vasculature case, implying that the vasculature does not merely decrease the strain throughout the brain but also causes a redistribution.

**FIGURE 9:**
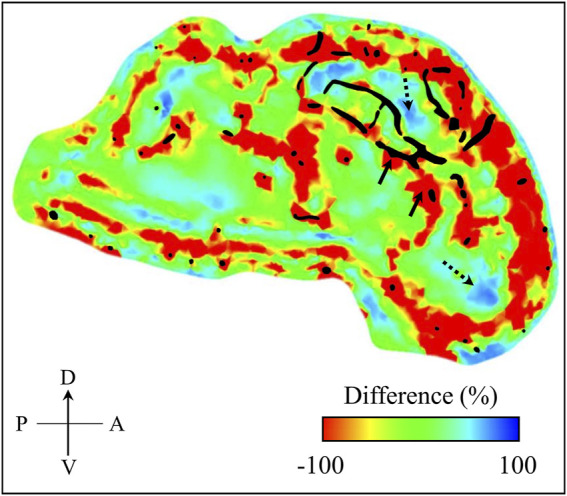
Percentage difference in maximum principal strain between the minipig-head model with and without vasculature. We computed this difference map over the mid-sagittal plane at time t = 4.36 ms into the simulation. For context, we superimposed the vasculature (represented by the black shaded region) over the mid-sagittal plane of the brain geometry used in our model. Through the difference map, we observed both decrease (indicated by solid-line arrows) and increase (indicated by dotted-line arrows) of maximum principal strain in the brain. A: anterior; D: dorsal; P: posterior; V: ventral.

To confirm this conjecture, we computed the percentage difference of the maximum principal strain between the models with and without vasculature at time t = 4.36 ms using EnSight 10.2.5a (ANSYS Inc., Canonsburg, PA). From the difference map, we observed a strain reduction of ∼ 100% in regions close to the vasculature indicated by the black-shaded color in Figure 9. Concurrently, we also observed a few regions along the mid-sagittal plane where the strain increased by 40–70%, highlighting that the vasculature induces a redistribution rather than just a reduction of the strain in the brain tissue. We reported similar observations in our previous study using a human-head model, where we evaluated the effect of the vasculature on the strain and stress responses of brain tissues due to a blunt impact to the head (Subramaniam et al., 2021). Specifically, we showed that the vasculature influenced the redistribution of the stress and strain in the brain tissues by as much as 30%. Although the volume redundancy that occurs when using the embedded-element technique may influence these differences in strain, we believe this effect is minimal because this procedure adds less than 1% to the mass of the brain.

### Study Limitations

Our study has limitations. First, because the resolution of the µCT was 35 μm, we could not capture vessels with diameters less than 35 µm in our model. In addition, we assigned a uniform thickness of 0.054 mm (based on our measurement of the middle cerebral artery) to all the vessels in the vasculature model and did not differentiate between arteries and veins. Such simplification might influence the deformation of the brain due to blast loading and, in turn, the strain predictions. However, we believe that the comparative results between models with and without vasculature are valid. Second, due to the complex geometry of the vasculature, we did not explicitly model it in the brain. Instead, following the methodology used in our previous studies (Unnikrishnan et al., 2019; Subramaniam et al., 2021), we coupled the vasculature and the head model using the embedded-elements technique in Abaqus. This method increases the mass and, thereby, the stiffness of the FE model due to volume redundancy (Garimella and Kraft, 2017). While we cannot determine the increase in stiffness of the brain due to the added mass, we believe that the stiffening response observed for the model with vasculature stems from the significantly stiffer vasculature. Third, we investigated the biomechanical responses in the brain only for the first 5 ms of the blast-wave propagation. While we acknowledge that the maximum principal strain in the brain may evolve further after this initial period, the objective of our study was to characterize the biomechanical responses in the brain resulting from the initial interaction of the blast wave with the head, which potentially leads to, the so-called, primary blast injury. Nevertheless, we believe that our finding that the addition of vasculature to the brain induces a strain redistribution will remain valid after the first 5 ms of the blast-wave propagation. Finally, we discarded 22 pressure measurements from our analysis, either due to sharp negative or positive spikes in the temporal pressure profiles or due to pressure-sensor malfunction, and used only eight measurements (four measurements each for two sensor locations) for validating the model predictions.

## Conclusion

We performed experiments to develop a 3-D high-fidelity FE model of a Göttingen minipig head and to characterize the biomechanical responses on the brain tissue due to a blast exposure of 210 kPa, using a laboratory shock tube. The first set of experiments allowed us to obtain the geometry of the cerebral vasculature and to characterize the responses of brain tissues and the vasculature to high strain rates typical of a blast exposure. Using these data, we developed a 3-D FE model of the minipig’s head and validated it in a set of shock-tube experiments by comparing measured and predicted intracranial pressures. Our comparisons yielded a good agreement, with differences in maximum pressure of less than 6% at the two measured locations. As expected, our study showed that the vasculature influenced the predicted maximum principal strain but not the pressure. Indeed, incorporation of the vasculature in the model induced a strain redistribution, with an approximate 100% decrease in strain in regions near the interface between the vasculature and the brain tissue and an approximate 40–70% increase in regions along the mid-sagittal plane of the brain. The high-fidelity FE model developed in this work will help establish correlates between observed brain injuries and predicted biomechanical responses in a minipig and facilitate the development and validation of scaling laws to project observed injuries in an animal brain to the human brain.

## Data Availability

The datasets presented in this article are not readily available because a written request to the corresponding author along with a summary of the planned research are required to obtain the datasets and related analyses. Requests to access the datasets should be directed to jaques.reifman.civ@mail.mil.

## Author Disclaimer

The opinions and assertions contained herein are the private views of the authors and are not to be construed as official or reflecting the views of the U.S. Army, the U.S. Department of Defense (DoD), or The Henry M. Jackson Foundation for the Advancement of Military Medicine, Inc. (HJF). Any citations of commercial organizations and trade names in this report do not constitute an official U.S. Army, DoD, or HJF endorsement of approval of the products or services of these organizations. This paper has been approved for public release with unlimited distribution.
